# Integrating systemic immune-inflammation index, fibrinogen, and T-SPOT.TB for precision distinction of active pulmonary tuberculosis in the era of mycobacterial disease research

**DOI:** 10.3389/fmicb.2024.1382665

**Published:** 2024-04-25

**Authors:** Zhikang Yu, Zifang Shang, Qingyan Huang, Feiqiu Wen, Sandip Patil

**Affiliations:** ^1^Research Experiment Center, Meizhou People's Hospital, Meizhou Academy of Medical Sciences, Meizhou, China; ^2^Guangdong Engineering Technological Research Center of Clinical Molecular Diagnosis and Antibody Drugs, Meizhou, China; ^3^Department of Haematology and Oncology, Shenzhen Children’s Hospital, Shenzhen, China; ^4^Paediatric Research Institute, Shenzhen Children’s Hospital, Shenzhen, China

**Keywords:** tuberculosis diagnosis, systemic immune-inflammation index (SII), fibrinogen, T-SPOT.TB, pulmonary disease differentiation

## Abstract

**Background:**

The clinical challenge of differentiating suspected tuberculosis with positive T-SPOT.TB results persist. This study aims to investigate the utility of the Systemic Immune-Inflammation Index (SII), Fibrinogen, and T-SPOT.TB in distinguishing between active pulmonary tuberculosis (PTB) and non-tuberculous lung diseases.

**Methods:**

A retrospective analysis included 1,327 cases of active PTB with positive T-SPOT.TB results and 703 cases of non-tuberculous lung diseases from May 2016 to December 2020 at Meizhou People’s Hospital. These were designated as the case group and the control group, respectively. The detection indicators of T-SPOT.TB: Early Secreted Antigenic Target 6 (ESAT-6), Culture Filtrate Protein 10 (CFP-10), as well as SII and Fibrinogen levels—were compared and analyzed for association and joint diagnostic value between the two groups.

**Results:**

The case group showed higher values of ESAT-6, CFP-10, SII, and Fibrinogen compared to the control group (all *p* < 0.001). In the case group, SII and Fibrinogen did not correlate with ESAT-6 and CFP-10 (∣rs∣ all < 0.3) but were positively correlated with C-reactive protein (CRP; rs all > 0.3). SII and Fibrinogen values in smear-positive pulmonary tuberculosis were higher than in smear-negative cases (all *p* < 0.05). The optimal diagnostic thresholds for ESAT-6, CFP-10, SII, and Fibrinogen in differentiating between active PTB and non-tuberculous lung diseases were 21.50 SFCs/10^6^ PBMC, 22.50 SFCs/10^6^ PBMC, 2128.32, and 5.02 g/L, respectively. Regression logistic analysis showed that ESAT-6 < 21.5 (OR: 1.637, 95% CI: 1.311–2.043, *p* < 0.001), CFP-10 < 22.5 (OR: 3.918, 95% CI: 3.138–4.892, *p* = 0.025), SII < 2128.32 (OR: 0.763, 95% CI: 0.603–0.967, *p* < 0.001), and FIB < 5.02 (OR: 2.287, 95% CI: 1.865–2.806, *p* < 0.001) were independent risk factors for active PTB. The specificity for ESAT-6 + CFP-10, ESAT-6 + CFP-10 + SII, ESAT-6 + CFP-10 + FIB, and ESAT-6 + CFP-10 + SII + FIB was 82.5%, 83.2%, 95.8%, and 80.1%, respectively, while sensitivity was 52.6%, 53.0%, 55.8%, and 44.7%, and positive predictive values were 85.0%, 85.6%, 84.1%, and 89.6%, respectively.

**Conclusion:**

SII and Fibrinogen are positively correlated with the degree of tuberculosis inflammation and the bacterial load of *Mycobacterium tuberculosis*. The combined detection of SII, Fibrinogen, and T-SPOT.TB is significant in distinguishing between active PTB with positive T-SPOT.TB results and non-tuberculous lung diseases.

## 1 Introduction

Tuberculosis, caused by *Mycobacterium tuberculosis* (Mtb) remains globally prevalent and high mortality rate, ranking second only to COVID-19 (Kaufmann, 2023). Prompt diagnosis and treatment, especially for pulmonary tuberculosis, are critical for patient care and curbing transmission (Pang et al., 2019). In China, only approximately 30% of pulmonary tuberculosis cases undergo confirmation through pathogen testing, posing challenges in differentiating active pulmonary tuberculosis (ATB) from non-tuberculous pulmonary diseases with similar radiographic features (Yang and Gao, 2018). Traditional diagnostic methods, relying on smear microscopy and culture, suffer from prolonged duration and low detection rates for negative cases. Nucleic acid detection techniques, while sensitive and rapid, present challenges such as high cost and complex operation, leading to prolonged evaluation times and increased financial burdens (Sun et al., 2022). The World Health Organization (WHO) encourages the development of non-sputum specimen diagnostic techniques, with a growing trend toward combining multiple markers (Verma et al., 2024). Early secreted antigen target 6 (ESAT-6), a protein specific to *Mycobacterium tuberculosis*, and the culture filtrate protein 10 (CFP-10) are indicative of tuberculosis infection, detectable through the tuberculosis infection T-cell spot test (T-SPOT.TB; Hamada et al., 2021; Ortiz-Brizuela et al., 2023). The WHO recommends T-SPOT.TB is primarily for the diagnosis of latent tuberculosis infection (LTBI). In immunocompetent individuals, T-SPOT.TB can differentiate between active and non-active tuberculosis, but a higher detection threshold is often required to identify active pulmonary tuberculosis (APTB; Yang et al., 2020). Hence, there remains a necessity for refined protocols in the utilization of T-SPOT.TB for the differential diagnosis of active tuberculosis. The Systemic Immune-Inflammation Index (SII), gauging immunity and inflammation based on platelet, neutrophil, and lymphocyte counts, serves as a comprehensive biomarker widely used in predicting cancer incidence and assessing inflammatory states (Nøst et al., 2021; Hamad et al., 2022). It reflects the individual’s immune status against pathogen infections and the severity of the disease, with minimal influence from mixed conditions (Li et al., 2023), rendering it a novel immune-inflammatory marker that has garnered considerable attention. Previous studies have indicated an association between elevated SII and tuberculosis, with a correlation observed with anxiety (Liu et al., 2022). Fibrinogen, a coagulation system activator, shows heightened levels in tuberculosis infections (Kager et al., 2015; Pinelo et al., 2023). Their roles in the differential diagnosis of suspected tuberculosis remain underexplored. The relationship between SII and Fibrinogen with Mtb infection has been initially comprehended, yet their involvement in the differential diagnosis of suspected tuberculosis has been rarely reported. Hence, this study retrospectively analyzes the T-SPOT.TB, SII, and Fibrinogen test values in 1,327 patients with active pulmonary tuberculosis (APTB) presenting positive T-SPOT.TB results, along with 703 patients exhibiting common clinical non-tuberculosis pulmonary diseases (non-TB). The aim is to explore their collective diagnostic value in the diagnosis and treatment of pulmonary tuberculosis.

## 2 Materials and methods

This study was conducted in accordance with the guidelines of the Declaration of Helsinki and approved by the Institutional Ethics Committee of our hospital, Meizhou People’s Hospital reference number: (2021-C-120) which complies with international ethical standards. Written consent was waived as the research did not use personal information, including names, for research purposes, and data were treated confidentially in line with the principles of the Declaration of Helsinki.

### 2.1 Subjects

**Figure 1:**
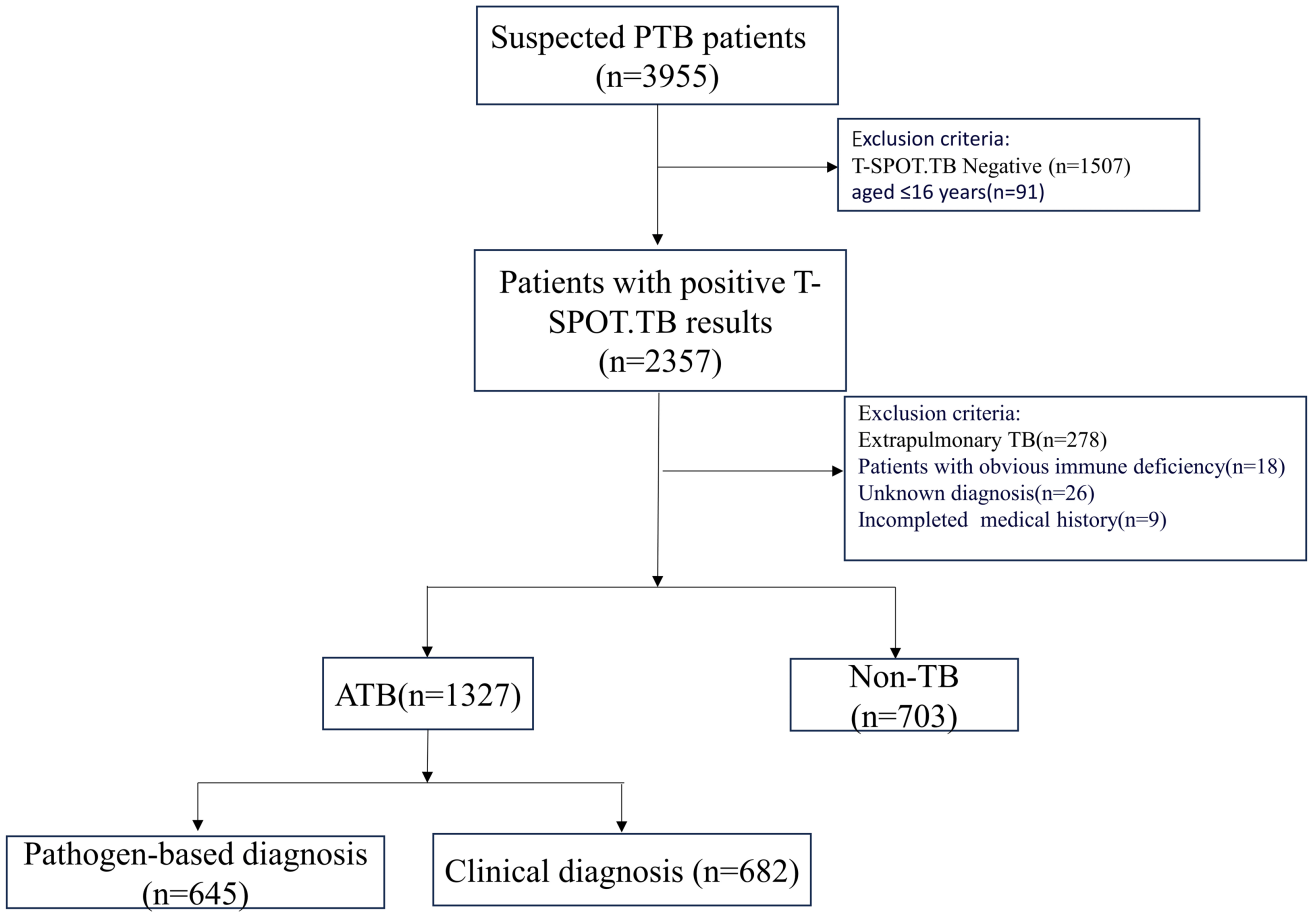
Flowchart of study data.

A total of 1,327 patients were diagnosed with active pulmonary tuberculosis (APTB) at Meizhou People’s Hospital from May 2016 to December 2020. During the same period, 703 non-TB patients were selected as the control group. APTB patients were identified based on the criteria outlined in the “WS 288-2017 Diagnosis of Pulmonary Tuberculosis,” According to this guideline, tuberculosis lesions occur in the lungs, trachea, bronchi, and pleura, these cases including 645 confirmed cases with positive sputum smears, cultures, or *Mycobacterium tuberculosis* nucleic acid tests, and 682 clinically diagnosed cases with evidence of clinical symptoms, signs, imaging findings, and effective tuberculosis treatment. Cases of extrapulmonary tuberculosis without pulmonary tuberculosis and old pulmonary tuberculosis were excluded. The definition of old tuberculosis is as follows: Firstly, patients with a history of tuberculosis must have recovered; secondly, despite the presence of pathological changes revealed by radiological diagnosis, no symptoms of tuberculous poisoning are observed; and thirdly, the results of etiology and pathology tests must be negative. Non-TB patients were those who were diagnosed with pulmonary diseases, excluding PTB, and had evidence such as pneumonia (Supplementary Figure S1). Both groups of patients were aged ≥ 16 years. After T-SPOT.TB testing and blood cell analysis, cases with negative or indeterminate T-SPOT.TB test results were excluded, as well as cases with obvious immune deficiency diseases such as acquired immune deficiency syndrome (HIV) etc. (Figure 1).

### 2.2 T-SPOT.TB detection

The T-SPOT.TB test kit (Oxford Immunotech, United Kingdom) was utilized according to the following steps: (1) Peripheral venous blood collection; (2) Isolation, collection, and counting of peripheral blood mononuclear cells (PBMCs); (3) Incubation of PBMCs with antigens (ESAT-6 as antigen A and CFP-10 as antigen B) for 16–20 h; (4) Spot counting; and (5) Result interpretation (Meier et al., 2005). The results are analyzed according to the following criteria: The detection result was positive when the number of spots in the blank control hole was 0 to 5 and (the number of spots in antigen A or antigen B hole—the number of blank control hole) ≥6, or the number of spots in blank control hole was 6 to 10 and (the number of spots in antigen A or antigen B hole) ≥2 × (the number of spots in blank control hole); If the above criteria are not met and the positive control hole is normal, the test result is negative.

### 2.3 Collection and examination of blood samples

After an overnight fast of 8–12 h, blood samples were collected from the anterior cubital vein using venipuncture. The collection tubes contained EDTA-K2 anticoagulant (2 mL), sodium citrate anticoagulant (2 mL), and gel progel (5 mL). Standard operating procedures on LBY-XC40B, AU5400, ACLTOP700, and SYSMEX XE-2100 blood analyzers were employed to examine erythrocyte sedimentation rate (ESR), C-reactive protein (CRP), four blood coagulation indexes, and blood cell composition, respectively. These included white blood cell count (WBC), neutrophils, lymphocytes, monocytes, platelets, red blood cell count (RBC), lymphocyte hemoglobin (HGB), prothrombin time (PT), activated partial thromboplastin time (APTT), thrombin time (TT), and fibrinogen (FIB).

### 2.4 Data processing and statistical analysis

With APTB as the target disease and non-tuberculous pulmonary disease as the control, the following indicators were used to evaluate its clinical diagnostic efficacy: sensitivity = True positive cases/(true positive cases + false negative cases), specificity = true negative cases/(true negative cases + false positive cases), positive predictive value = True positive cases/(true positive cases + false positive cases), negative predictive value = true negative cases/(true negative cases + false negative). SPSS 21.0 and GraphPad Prism 8.0 were used for data analysis and mapping, and continuous data were compared using the T-test or Mann–Whitney *U*-test. The categorical variables were expressed numerically (%) and compared using the Chi-square test or Fisher exact test. Pearson and Spearman correlation analysis analyzed the relationship between the correlation test indicators, with the correlation coefficient being denoted by rs. Binary Logistic regression analysis was employed to assess the clinical indicators that influence the occurrence of active pulmonary tuberculosis. The relative risk was quantified using the Odds Ratio (OR) along with its corresponding 95% Confidence Interval (CI). Furthermore, the new value for each combination was derived from the pertinent binary logistic regression analysis equation. A Delong test was employed to assess the statistical significance among the AUCs. ROC curve analysis was conducted to determine the optimal cutoff values of ESAT-6, CFP-10, SII, FIB, ESAT-6 + CFP-10, ESAT-6 + CFP-10 + SII, ESAT-6 + CFP-10 + FIB, and ESAT-6 + CFP-10 + MLR + PLR for distinguishing APTB from non-TB. The area under the curve (AUC) and cutoff values were determined with the highest sum of sensitivity and specificity. The sensitivities, specificities, and positive and negative predictive values of each threshold were calculated. The 95% confidence intervals (95%CI) were calculated using the Wilson score method. *p* < 0.05 indicated a statistically significant difference.

## 3 Results

### 3.1 General information and laboratory parameters

A total of 2,030 cases were enrolled in the Hakka area of South China, including 1,327 cases (1,081 males and 246 females) in the case group, with an average age of (60.48 ± 15.83) years. There were 703 cases (548 males and 155 females) in the control group, with an average age of (61.52 ± 13.23) years, and the age of the case group was lower than that of the control group. The levels of PT, TT, leukocytes, neutrophils, and lymphocytes in the case group were lower than those in the control group (*p* < 0.05). The levels of PLT, FIB, SII, CRP, ESR, ESAT-6, and CFP-10 in the case group were higher than those in the control group (*p* < 0.05). The levels of SIRI, HB, and APPT in the case group were lower than those in the control group, and the monocyte counts and RBC in the case group were higher than those in the control group, but the differences were not statistically significant (Table 1).

**Table 1:** Characteristics and laboratory parameters of patients with pulmonary tuberculosis and non-tuberculous pulmonary disease.

Parameter	Non-tuberculosis (*n* = 703)	Pulmonary tuberculosis (*n* = 1,327)	t/U	*p*-values
**Sex**				
Male	548(78.0%)	1,081 (81.5%)		
Female	155(22.0%)	246 (18.5%)		
Age(year)	61.52 ± 13.23	60.48 ± 15.83	*t* = −1.489	0.137
WBC(×10^12^/L)	11.97(7.20,14.10)	9.70(6.60,11.60)	U = −6.95	<0.001
Neutrophil (×10^9^/L)	8.85(4.90,11.20)	7.54(4.60,9.00)	*U* = −5.478	<0.001
Lymphocyte(×10^9^/L)	2.00(0.90,2.00)	1.28(0.80,1.60)	*U* = −7.018	<0.001
Monocytes (×10^9^/L)	0.97(0.40,1.00)	0.74(0.50,0.90)	U = −0.196	0.845
RBC(×10^12^/L)	4.25 ± 1.07	4.31 ± 0.79	*t* = −1.316	0.188
HB(g/L)	121.92 ± 29.46	121.46 ± 22.55	*t* = 1.237	0.216
PLT(×10^9^/L)	223.33(148.00,274.00)	298.83(210.00,367.00)	U = −13.848	<0.001
SII	1697.2(559.09,2185.84)	2440.11(935.72,2789.44)	U = −8.571	<0.001
SIRI	6.44(1.62,7.50)	6.35(1.98,6.90)	U = −1.308	0.191
PT(S)	13.44 ± 4.36	12.96 ± 2.76	*t* = 2.699	0.008
TT(S)	15.04 ± 4.37	14.66 ± 2.59	*t =* 2.122	0.034
APTT(S)	34.04 ± 8.47	33.95 ± 5.78	*t* = 0.225	0.822
FIB(g/L)	5.01 ± 1.89	5.66 ± 1.75	*t* = −7.583	<0.001
ESR(mm/h)	40.26(11.0,60.0)	48.48(19.0,75.0)	*U* = −6.938	<0.001
CRP(mg/L)	51.62(8.76,84.29)	54.20(11.55,84.37)	U = −1.977	0.048
ESAT-6	18.64(7.0,22.0)	38.06(7.0,46.0)	U = −8.727	<0.001
CFP-10	17.70(5.0,21.0)	60.04(10.0,73.0)	*U* = −16.120	<0.001

SII = neutrophil*platelet to lymphocyte ratio, SIRI = neutrophil*monocyte to lymphocyte ratio.

### 3.2 Receiver operating characteristic curve analysis results of each test index used in the differential diagnosis of suspected TB

**Figure 2:**
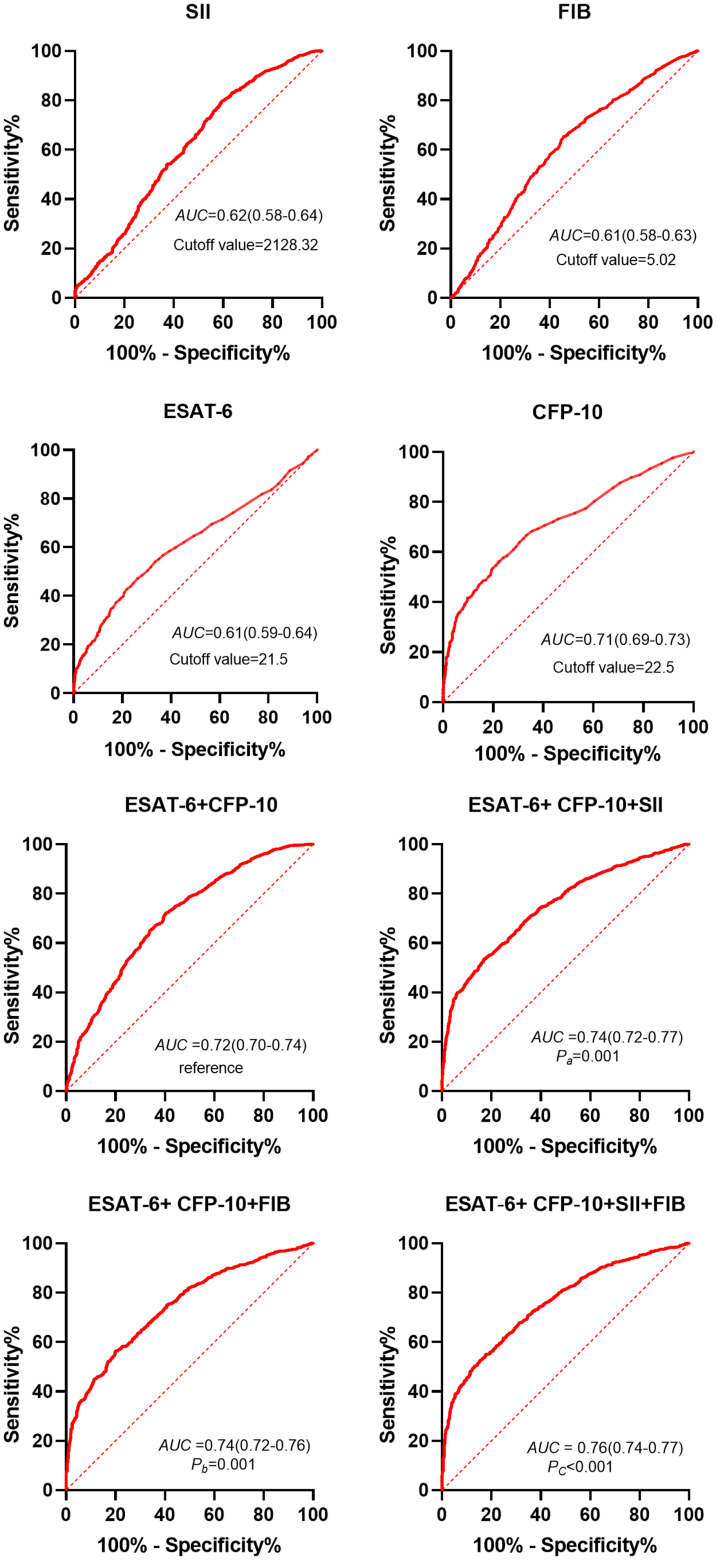
ROC analysis results of different indexes in patients with pulmonary tuberculosis and non-tuberculous lung disease with positive T-SPOT results. Dotted line, curvature response sensitivity index of ROC curve; Red line, receiver Operating Characteristic Curve; AUC, Area under ROC Curve; ESAT-6, early secretory antigen target 6; CFP-10, culture filtrate protein 10; SII, systemic immune-inflammation index. *P_a_*: [ESAT-6 + CFP-10 + SII] vs. [ESAT-6 + CFP-10]; *P_b_*: [ESAT-6 + CFP-10 + FIB] vs. [ESAT-6 + CFP-10]; *P_c_*:[ESAT-6 + CFP-10 + FIB+SII] vs. [ESAT-6 + CFP-10].

The optimal critical values of ESAT-6, CFP-10, SII, FIB, ESAT-6 + CFP-10, ESAT-6 + CFP-10 + SII, ESAT-6 + CFP-10 + FIB, and ESAT-6 + CFP-10 + SII + FIB is 21.5 SFCs/10^6^ PBMC (spot-forming cells, SFCs; Peripheral Blood Mononuclear Cell, PBMC), 22.5 SFCs/10^6^ PBMC, 2128.32, 5.02 g/L, 0.65, 0.68, 0.66, and 0.75, respectively, by receiver operating characteristic (ROC) curve analysis. Furthermore, the calculation of the new values for these combinations was based on the following formulae: y_[ESAT-6+ CFP-10]_ = 0.217 + 0.007*ESAT-6 + 0.023*CFP-10, y_[ESAT-6+ CFP-10 + SII]_ = −0.661 + 0.008*ESAT-6 + 0.024*CFP-10 + 0.001*SII, y_[ESAT-6+ CFP-10 + FIB]_ = −1.339 + 0.006*ESAT-6 + 0.023*CFP-10 + 0.211*FIB, and y_[ESAT-6+ CFP-10 + SII + FIB]_ = −1.521 + 0.008*ESAT-6 + 0.024*CFP-10 + 0.001*SII + 0.175*FIB. When the optimal thresholds are set, the ROC analysis shows ESAT-6, CFP-10, SII, FIB, ESAT-6 + CFP-10, ESAT-6 + CFP-10 + SII, ESAT-6 + CFP-10 + FIB, ESAT-6 + CFP-10 + SII + FIB (area under curve, AUC) were 0.61, 0.71, 0.62, 0.61, 0.72, 0.74, 0.74, 0.76, respectively. Taking the AUC of T-SPOT.TB detection [ESAT-6+ CFP-10] as a reference, the comparisons of the AUCs of diverse combinations against this reference revealed significant differences, with *P_a_* equaling 0.001, *P_b_* equaling 0.001, and *Pc* being less than 0.0001 (Figure 2).

### 3.3 Correlation between SII, FIB, and other detection indexes and relationship with tuberculosis disease degree

**Figure 3:**
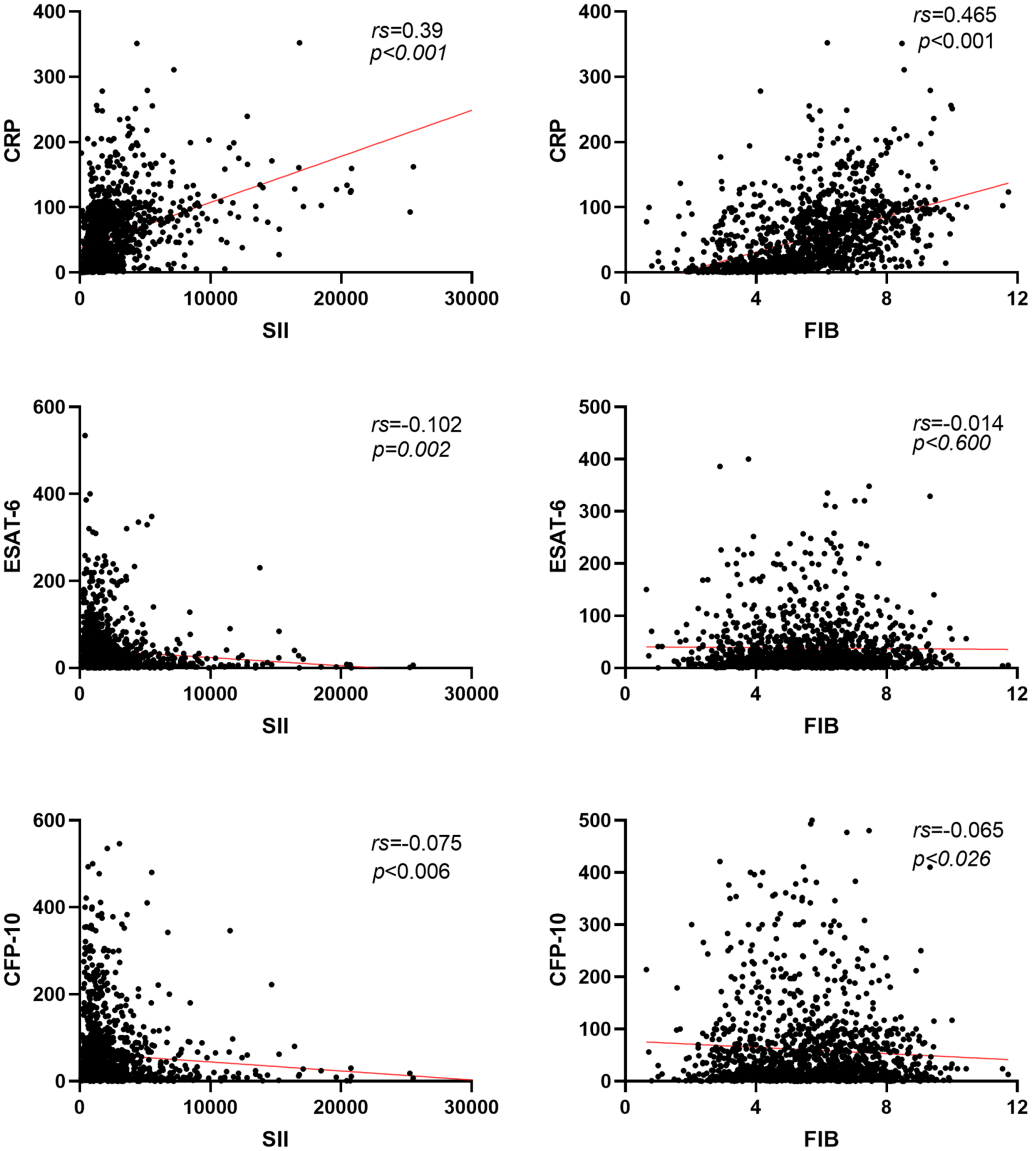
Correlation analysis of SII and FIB with ESAT-6, CFP-10, and CRP in patients with pulmonary tuberculosis. Red line, the linear trend line of the two detection indicators; rs, spearman rank correlation coefficient; CRP, C-reactive protein.

**Figure 4:**
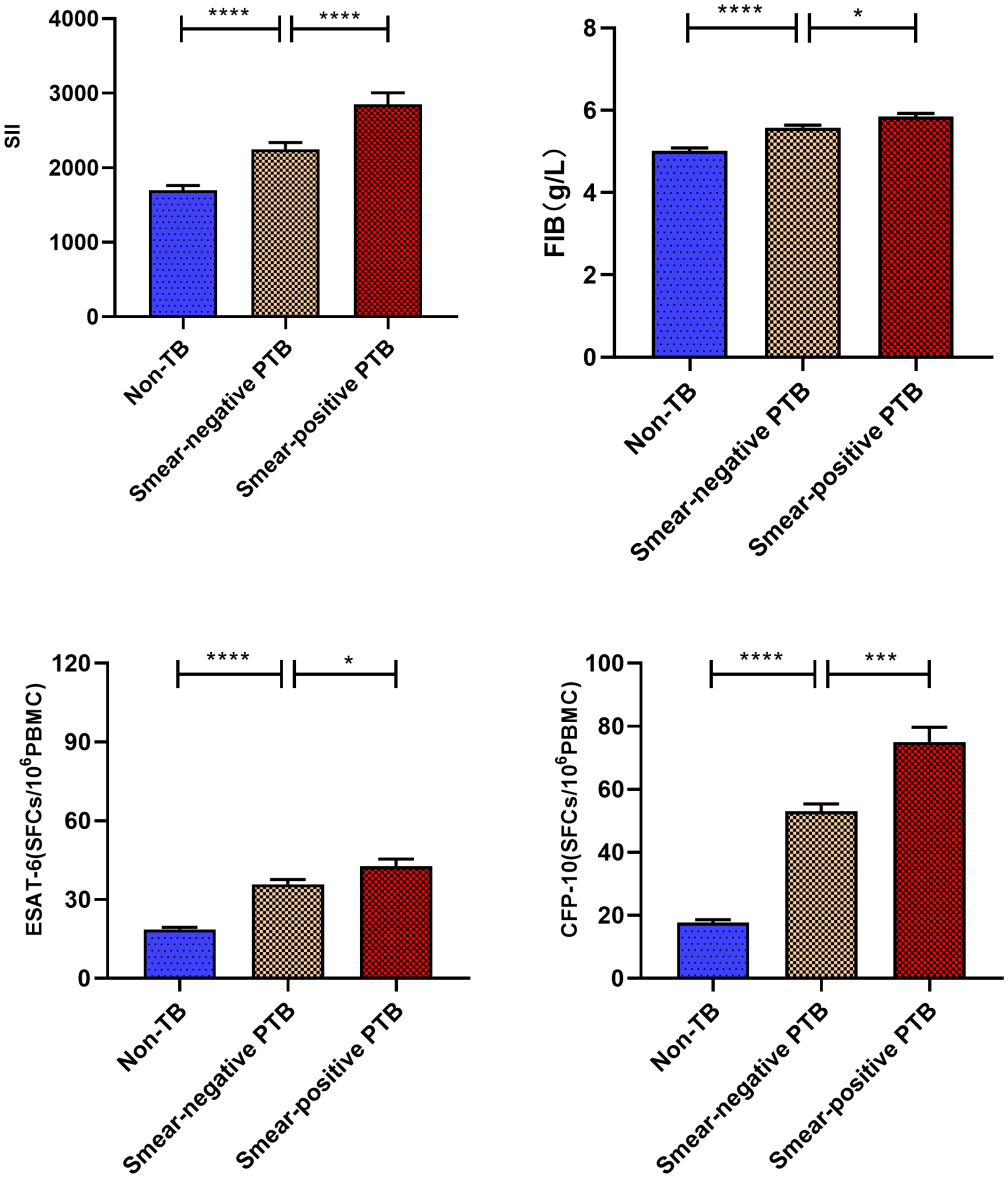
Comparative analysis of systemic immune index (SII) and FIB values in various groups. ^****^p < 0.0001, ^***^p < 0.001, ^*^p < 0.05. All data are presented as means ± SEMs.

Based on Figure 3, it can be observed that the Systemic Inflammation Index (SII, SII = neutrophil*platelet to lymphocyte ratio) and Fibrinogen (FIB) values of active pulmonary tuberculosis (APTB) patients are not correlated with ESAT-6 (rs = −0.102, −0.075) and CFP-10 (rs = −0.014, −0.065), with all ∣rs∣ values being <0.3. However, there is a positive correlation with C-reactive protein (CRP; rs = 0.390, 0.465), where all rs values are >0.3. As depicted in Figure 4, the levels of SII, FIB, ESAT-6, and CFP-10 in smear-positive pulmonary tuberculosis are significantly higher than those in smear-negative pulmonary tuberculosis (*p* < 0.0001, *p* < 0.05, *p* < 0.05, *p* < 0.001, respectively). Additionally, the SII, FIB, ESAT-6, and CFP-10 levels in the APTB group are significantly higher than those in the non-TB group.

### 3.4 Logistic regression analysis of risk factors of active pulmonary tuberculosis

Univariate analysis and multivariate logistic regression analysis were performed to measure the relationship between detection indicators and active pulmonary tuberculosis. The results of univariate analysis showed that ESAT-6 < 21.5 (OR: 2.500, 95% CI: 2.048–3.052, *p* < 0.001), CFP-10 < 22.5 (OR: 4.443, 95% CI: 3.609–5.468, *p* < 0.001), SII < 2128.32 (OR: 0.834, 95% CI: 0.674–1.032, *p* = 0.095), and FIB<5.02 (OR: 2.243, 95% CI: 1.861–2.703, *p* < 0.001) were significantly associated with active pulmonary tuberculosis. Multivariate regression logistic analysis showed that ESAT-6 < 21.5 (OR: 1.637, 95% CI: 1.311–2.043, *p* < 0.001), CFP-10 < 22.5 (OR: 3.918, 95% CI: 3.138–4.892, *p* = 0.025), SII < 2128.32 (OR: 0.763, 95% CI: 0.603–0.967, *p* < 0.001), and FIB<5.02 (OR: 2.287, 95% CI: 1.865–2.806, *p* < 0.001) were independent risk factors for active pulmonary tuberculosis (Table 2). ESAT-6 < 21.5, CFP-10 < 22.5, SII < 2128.32, and FIB < 5.02 can be used to diagnosis of active pulmonary tuberculosis.

**Table 2:** Logistic regression analysis of risk factors of active pulmonary tuberculosis.

Variables	Univariate		Multivariate	
	OR (95% CI)	*p*-value	OR (95% CI)	*p*-value
Gender (female/male)	0.805 (0.642–1.008)	0.059	1.209 (0.979–1.493)	0.079
Age (<60/≥60, years old)	0.844 (0.700–1.018)	0.076	0.797 (0.622–1.023)	0.074
ESAT-6 (<21.5/≥21.5)	2.500 (2.048–3.052)	<0.001	1.637 (1.311–2.043)	<0.001
CFP-10 (<22.5/≥22.5)	4.443 (3.609–5.468)	<0.001	3.918 (3.138–4.892)	<0.025
SII (<2128.32/≥2128.32)	0.834 (0.674–1.032)	<0.095	0.763 (0.603–0.967)	<0.001
FIB (<5.02/≥5.02)	2.243 (1.861–2.703)	<0.001	2.287 (1.865–2.806)	<0.001

OR, odds ratio; CI, confidence interval.

### 3.5 The value of different indexes and their combined detection in the differential diagnosis of suspected TB

As shown in Table 3, When the optimal thresholds are set, the specificity of ESAT-6, CFP-10, SII, FIB, ESAT-6 + CFP-10 + SII, ESAT-6 + CFP-10 + FIB, ESAT-6 + CFP-10 + SII + FIB in the differential diagnosis of APTB and non-tuberculosis pulmonary disease was 73.7%(95%CI 70.4–76.9), 77.5%(95%CI 74.4–80.6), 54.3%(95%CI 51.4–58.8), 82.5%(95%CI 79.7–85.3), 83.2%(95%CI 80.4–85.9), 80.1%(95%CI 77.1–83.0), 90.2%(95%CI 87.9–92.4), respectively. The positive predictive values was77.2% (95%CI 74.3–80.1), 82.5% (95%CI 80.0–85.0), 72.4% (95%CI 68.8–75.7), 72.9% (95%CI 71.1–76.1), 85.0% (95%CI 82.5–87.5), 85.6% (95%CI 83.2–88.0), 84.1% (95%CI 81.7–86.5), 89.6% (95%CI 87.2–91.9), respectively.

**Table 3:** The diagnostic efficacy of different indexes in patients with pulmonary tuberculosis and non-tuberculosis pulmonary disease with positive T-SPOT.TB results.

Indicators	Sensitivity (95%CI)	Specificity (95%CI)	Positive predictive value(95%CI)	Negative predictive value(95%CI)	AUC(95%CI)
ESAT-6	47.2%(44.5–49.8)	73.7%(70.4–76.9)	77.2%(74.3–80.1)	42.4% (39.7–45.3)	0.61(0.59–0.64)
CFP-10	56.3%(53.6–58.9)	77.5%(74.4–80.6)	82.5%(80.0–85.0)	48.4%(45.5–51.4)	0.71 (0.69–0.73)
SII	35.5%(32.9–38.0)	74.4%(71.1–77.5)	72.4%(68.8–75.7)	37.9%(35.4–40.5)	0.62 (0.58–0.64)
FIB	65.3%(62.7–67.8)	54.3%(51.4–58.8)	72.9%(71.1–76.1)	45.4%(41.9–48.7)	0.61 (0.58–0.63)
ESAT-6 + CFP-10	52.6%(49.9–55.3)	82.5%(79.7–85.3)	85.0%(82.5–87.5)	47.9%(45.2–50.8)	0.72 (0.70–0.74)
ESAT-6 + CFP-10 + SII	53.0%(50.3–55.6)	83.2%(80.4–85.9)	85.6%(83.2–88.0)	48.4%(45.5–51.2)	0.74 (0.72–0.77)
ESAT-6 + CFP-10 + FIB	55.8%(53.1–58.5)	80.1%(77.1–83.0)	84.1%(81.7–86.5)	49.0%(46.1–51.9)	0.74 (0.72–0.76)
ESAT-6 + CFP-10 + SII + FIB	44.7%(42.0–47.4)	90.2%(87.9–92.4)	89.6%(87.2–91.9)	46.3%(43.7–48.9)	0.76 (0.74–0.77)

## 4 Discussion

The clinical characteristics and imaging results of active pulmonary tuberculosis (APTB) and non-tuberculous lung diseases are similar, making it challenging to distinguish between the two. Early diagnosis of tuberculosis is difficult, leading to many undiagnosed cases, contributing to the sustained transmission of tuberculosis and an increased disease burden (Calderwood et al., 2023; Verma et al., 2024). With the widespread application of T-SPOT.TB testing, various approaches have been provided for its auxiliary diagnosis of active tuberculosis. Studies indicate that raising the T-SPOT.TB detection threshold has diagnostic value for active tuberculosis (Calderwood et al., 2023; Verma et al., 2024). Combining T-SPOT.TB with other meaningful indicators can differentiate suspected pulmonary tuberculosis patients (Liu et al., 2021). *Mycobacterium tuberculosis* infection can induce changes in hematopoietic stem cell proliferation and immune responses, leading to alterations in lymphocyte, platelet, and other cell proportions (Kirwan et al., 2021; Chen et al., 2022). SII is a comprehensive new immunoinflammatory biomarker based on platelet, neutrophil, and lymphocyte counts. Changes in the proportions of peripheral blood cells it represents can accurately reflect local immune responses and systemic inflammatory status (Zhao et al., 2023). Elevated fibrinogen levels may be associated with lung damage caused by tuberculosis infection (Dong et al., 2018). Known immune and coagulation-related indicators SII and FIB are associated with tuberculosis, but their role in distinguishing between APTB and non-TB diagnoses is unclear. This study explores their role in the differential diagnosis between the two and finds that the SII and FIB levels in the APTB group are significantly higher than those in the non-TB group. Moreover, they are positively correlated with the degree of tuberculosis inflammation and related to the tuberculosis bacterial load. Through both univariate and multifactor regression analyses, they, having exceeded the cut-off level, have been identified as risk factors for active pulmonary tuberculosis. Simultaneously, the combination of SII and FIB with T-SPOT.TB testing demonstrates good diagnostic efficacy in differentiating between pulmonary tuberculosis and non-tuberculous lung diseases. T-SPOT.TB testing has diagnostic value for distinguishing between active pulmonary tuberculosis (APTB) and non-tuberculous lung diseases (non-TB; Zhang et al., 2017; Luo et al., 2020; Sun et al., 2022). Our research results indicate that the levels of ESAT-6, CFP-10, Systemic Immune-Inflammation Index (SII), and Fibrinogen (FIB) in the APTB group are significantly higher than those in the non-TB group. This suggests that after tuberculosis infection, SII and FIB may experience varying degrees of elevation, consistent with some previous research findings (Kager et al., 2015; Liu et al., 2022). Inflammatory and coagulation indices tend to increase to different extents after tuberculosis infection (Ştefanescu et al., 2021; Mustafa et al., 2022). However, in our statistical data, the Systemic Inflammation Response Index (SIRI) and Activated Partial Thromboplastin Time (APTT) did not show significant differences between the two groups, possibly due to our control group being related to other pulmonary diseases. When optimal cutoff values were applied for ESAT-6, CFP-10, SII, and FIB (Figure 2; Table 3), the Area Under the Curve (AUC), specificity, and positive predictive values for differentiating between APTB and non-TB were good, indicating their utility in the differential diagnosis of suspected tuberculosis. Correlation analysis showed that SII and FIB are not correlated with CFP-10 and ESAT-6, suggesting that SII and FIB are valuable supplements to T-SPOT.TB testing for diagnosing APTB. Both SII and FIB are associated with recognized inflammatory markers, indicating their involvement in the inflammatory process caused by *Mycobacterium tuberculosis* infection. By comparing the levels of SII and FIB in sputum-positive and sputum-negative tuberculosis, as well as non-TB cases, it was found that SII and FIB are positively correlated with the bacterial load of tuberculosis. SII and FIB may be associated with the severity of tuberculosis, potentially serving as predictors of the efficacy of anti-tuberculosis treatment, a topic that will be explored in future research. SII is an indicator of inflammation and immune response, and numerous studies have focused on its effective prognosis in various types of diseases associated with systemic inflammatory reactions (Fest et al., 2018). There are also many studies related to infectious diseases (Mazza et al., 2020). SII levels significantly decrease from a high position after anti-tuberculosis treatment, but the specific reasons and mechanisms are not explained (Ştefanescu et al., 2021). A higher SII is also a predictive factor for depression and anxiety in tuberculosis patients (Liu et al., 2022). SII may be a potential indicator for TB diagnosis. There is currently no research applying it to the auxiliary diagnosis of active pulmonary tuberculosis (APTB). We found that SII when applied to the auxiliary diagnosis of APTB, has good specificity and positive predictive values, especially when used in conjunction with T-SPOT.TB testing, where both specificity and positive predictive values are greater than 83%. Fibrinogen is a common coagulation marker involved in tissue damage and inflammation (Luyendyk et al., 2019) *Mycobacterium tuberculosis* infection is a process of chronic damage and inflammation, and the acute phase of infection can induce a hypercoagulable state in the body (Kager et al., 2015). Compared to the non-TB group, we found higher levels of Fibrinogen in the APTB group. When applied to the auxiliary diagnosis of APTB, it also exhibits good sensitivity and positive predictive values. When combined with T-SPOT.TB testing, both specificity and positive predictive values are greater than 80%. Finally, we explored the application of combined testing with SII, Fibrinogen, and T-SPOT.TB for the auxiliary diagnosis of APTB, achieving specificity and positive predictive values close to 90%.

Some studies suggest that when the T-SPOT.TB detection threshold exceeds 100 Spot-Forming Cells (SFCs), the specificity for diagnosing APTB exceeds 90%, but clinically, the proportion of people with T-SPOT.TB detection thresholds of less than 100 SFCs are higher. In our approach, the optimal thresholds for ESAT-6 and CFP-10 are 21.5 SFCs and 22.5 SFCs, respectively, addressing the challenge of T-SPOT.TB requires high thresholds to distinguish suspected tuberculosis and provide a new approach for the clinical diagnosis of APTB. There was no statistically significant difference in the positive rates of CFP-10 and ESAT-6 between the disease group and the active TB subgroup, however, there was a statistically significant difference when both ESAT-6 and CFP-10 were positive (Ma et al., 2019). Fox et al. (2007) found that CFP-10 can be used primarily to detect active tuberculosis. The inconsistency of these findings may be due to the heterogeneity of the study population, so more in-depth studies are needed in future work. This study has certain limitations. In the screening and diagnostic protocols for active tuberculosis, the WHO encourages cost-effective solutions, prioritizing non-sputum tests with high sensitivity and specificity. While our approach may have lower sensitivity, the chosen diagnostic indicators are easy to profile, clinically feasible, and represent a non-sputum test with high specificity and positive predictive values. Additionally, this study is single-center research, and due to the incomplete representativeness of the subjects, the results may have bias. Future studies will incorporate multi-center validation for appropriate verification.

## 5 Conclusion

SII and FIB are positively correlated with the degree of tuberculosis inflammation and the bacterial load of *Mycobacterium tuberculosis*. Without raising the T-SPOT.TB detection threshold, the combined detection of SII, FIB, and T-SPOT.TB can effectively distinguish between active pulmonary tuberculosis and non-tuberculous lung diseases when T-SPOT.TB results are all positive. This has significant implications for the differential diagnosis of suspected tuberculosis.

## Data availability statement

The original contributions presented in the study are included in the article/Supplementary material, further inquiries can be directed to the corresponding author.

## Ethics statement

This study was conducted in accordance with the guidelines of the Declaration of Helsinki and approved by the Institutional Ethics Committee of our hospital, Meizhou People’s Hospital reference number: (2021-C-120) which complies with international ethical standards. The studies were conducted in accordance with the local legislation and institutional requirements. Written informed consent for participation was not required from the participants or the participants’ legal guardians/next of kin in accordance with the national legislation and institutional requirements.

## Author contributions

ZY: Conceptualization, Data curation, Funding acquisition, Investigation, Methodology, Visualization, Writing – original draft, Writing – review & editing. ZS: Conceptualization, Investigation, Project administration, Visualization, Writing – original draft, Writing – review & editing. QH: Conceptualization, Data curation, Validation, Writing – original draft, Writing – review & editing. FW: Formal analysis, Funding acquisition, Supervision, Writing – original draft, Writing – review & editing. SP: Conceptualization, Formal analysis, Methodology, Resources, Writing – original draft, Writing – review & editing.

## Supplementary material

SUPPLEMENTARY FIGURE S1 Composition of non-TB patients. COPD, chronic obstructive pulmonary disease.
