# Supplementary figures and images for: Integrating systemic immune-inflammation index, fibrinogen, and T-SPOT.TB for precision distinction of active pulmonary tuberculosis in the era of mycobacterial disease research
Source: Front Microbiol. 2024 Apr 25;15:1382665. doi: 10.3389/fmicb.2024.1382665 (PMC11079184; doi:10.3389/fmicb.2024.1382665)

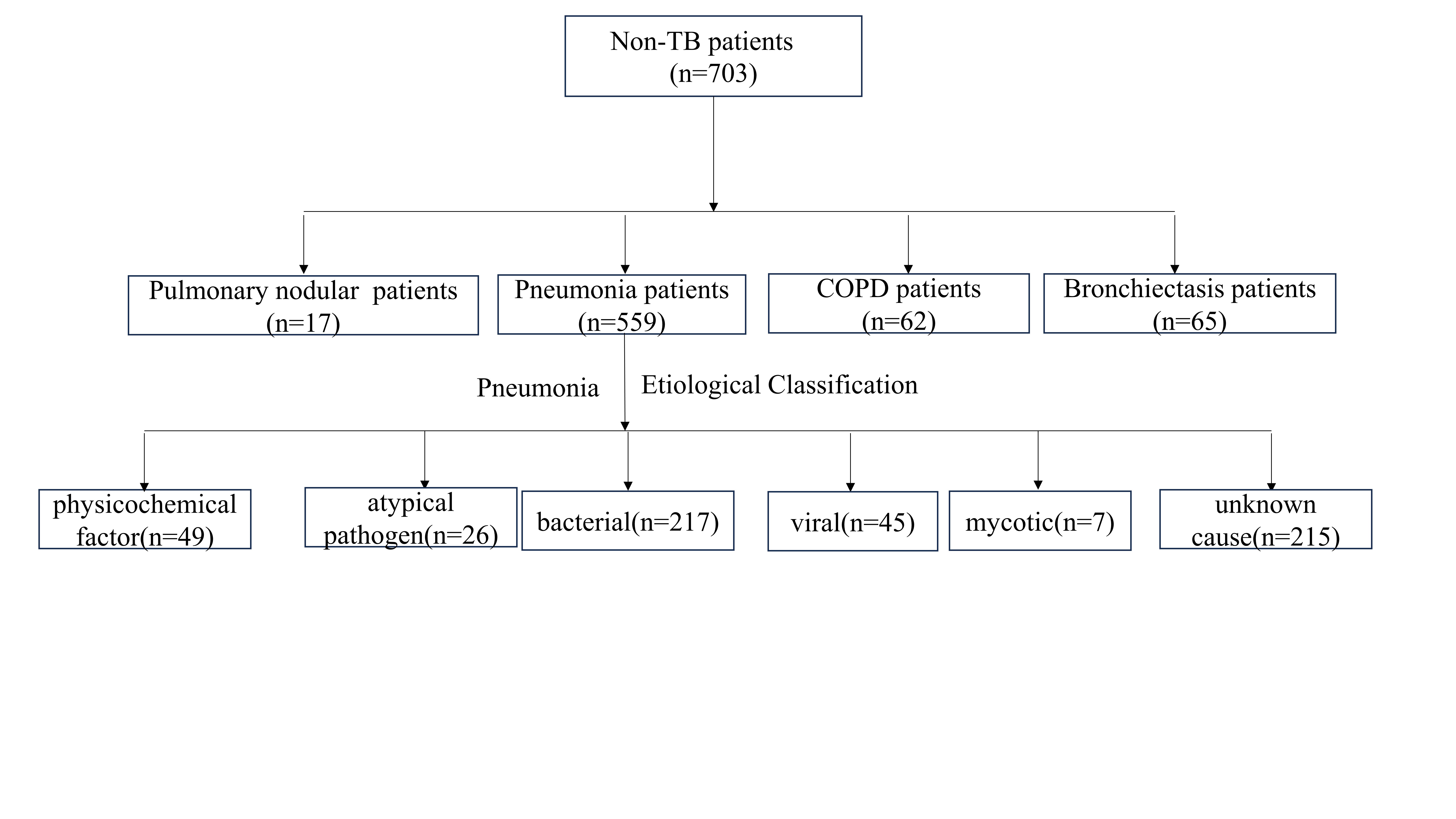

Supplement: SUPPLEMENTARY FIGURE S1 — Composition of non-TB patients. COPD, chronic obstructive pulmonary disease. [file Image_1.jpg]
